# Generalized Rash and Angioedema During Triple Antiepileptic Therapy: A Case Report

**DOI:** 10.7759/cureus.90700

**Published:** 2025-08-21

**Authors:** Zlatana K Perovic, Slavica Vujisic

**Affiliations:** 1 Neurology-Epileptology, General Hospital Niksic, Niksic, MNE; 2 Neurology-Epileptology, University of Montenegro Faculty of Medicine, Podgorica, MNE

**Keywords:** angioedema, antiepileptic drug, drug-induced rash, levetiracetam, pharmacoresistant epilepsy

## Abstract

We present the case of a 42-year-old woman with drug-resistant symptomatic focal epilepsy secondary to focal cortical dysplasia, first diagnosed at the age of 15. The patient was on triple antiepileptic drug (AED) therapy: levetiracetam (LEV), lamotrigine (LTG), and oxcarbazepine (OxCBZ). Three months after the introduction of oxcarbazepine, she developed a generalized skin rash accompanied by episodic angioedema, manifesting as lip swelling and intermittent dyspnea.

Oxcarbazepine was initially discontinued, as it was the most recently introduced drug, but the rash persisted. Lamotrigine was then withdrawn and replaced with lacosamide (LCZ), without significant improvement. Finally, levetiracetam was discontinued and substituted with zonisamide (ZON), resulting in the complete resolution of the cutaneous symptoms. The patient continued to experience two focal seizures per week, consistent with her baseline seizure frequency prior to medication adjustments.

This case underscores the importance of recognizing cutaneous adverse effects associated with AEDs and illustrates the therapeutic challenges in managing patients with pharmacoresistant epilepsy, particularly when adverse drug reactions complicate polytherapy.

## Introduction

Cutaneous adverse reactions occur in approximately 5%-10% of patients treated with antiepileptic drugs (AEDs) and represent a significant clinical challenge due to their potential severity, including life-threatening conditions such as Stevens-Johnson syndrome (SJS) and toxic epidermal necrolysis (TEN). These reactions are well-documented complications of several AEDs, including phenytoin, carbamazepine (CBZ), oxcarbazepine (OxCBZ), phenobarbital, primidone, zonisamide (ZON), and lamotrigine (LTG) [1].

These reactions range from diffuse, maculopapular, erythematous, pruritic, and urticarial rashes to severe hypersensitivity syndromes such as Stevens-Johnson syndrome (SJS), toxic epidermal necrolysis (TEN), and drug reaction with eosinophilia and systemic symptoms (DRESS) syndrome. Cutaneous symptoms typically develop within days to a few weeks after initiating the offending agent [2].

Angioedema can be mediated by IgE-dependent hypersensitivity or bradykinin-related mechanisms, both of which affect treatment approaches. Although less common, angioedema may also occur, either as part of a broader hypersensitivity reaction or as an isolated manifestation. These complications are particularly challenging in patients with pharmacoresistant epilepsy, where therapeutic flexibility is already limited [3].

Incorrectly identifying the causative AED in polytherapy settings may lead to unnecessary medication changes and prolonged exposure to the actual offending agent, which can result in persistent adverse effects and worsened patient outcomes [4]. Therefore, recognizing less typical culprits, such as levetiracetam (LEV), is essential in clinical practice to ensure timely intervention and optimize patient safety [5].

In this report, we present a case of a patient with long-standing pharmacoresistant focal epilepsy who developed a generalized rash and episodes of angioedema while receiving triple AED therapy. This case underscores the diagnostic complexity of adverse cutaneous drug reactions and the importance of careful sequential drug withdrawal in identifying the causative agent.

## Case presentation

Patient information

A 42-year-old woman with pharmacoresistant focal seizures secondary to focal cortical dysplasia (diagnosed at age 15) presented with a generalized rash persisting for one month. She denied any known drug allergies or relevant genetic conditions.

History of present illness

Her epileptic seizures began at the age of 15 as focal seizures, which manifested as an epigastric aura or a sudden feeling of fear lasting around 10 seconds, occasionally accompanied by speech arrest lasting 10-20 seconds. At that time, brain magnetic resonance imaging (MRI) revealed focal cortical dysplasia in the left parietotemporal region.

Her early psychomotor development was normal. She had no history of febrile seizures, other illnesses, or head injuries. Initially, seizures occurred 3-4 times per month, with occasional secondary generalized tonic-clonic seizures occurring once a month. The frequency of seizures was approximately once every 4-6 months. Initial treatment with sodium valproate (VPA), titrated up to 1500 mg/day, did not succeed in reducing the frequency of focal seizures. Topiramate (TPM) was added to the therapy and titrated up to 400 mg/day, which eliminated the generalized seizures, but focal seizures continued to occur at a frequency of 1-2 episodes per week.

Later, VPA was replaced with carbamazepine (CBZ) at 800 mg/day, but in combination with the existing TPM therapy, there was no change in seizure frequency. Four years ago, the seizure frequency increased, with seizures occurring daily, sometimes 2-3 times per day. The semiology of the seizures changed (e.g., numbness on the right side of the body, prolonged speech arrest, and dizziness). At that time, TPM was replaced with lamotrigine (LTG) at 500 mg/day, which resulted in a significant reduction in seizure frequency to 1-2 per month. She even experienced seizure-free periods lasting 2-3 months.

Eight months ago, CBZ was replaced with levetiracetam (LEV) at 3000 mg/day, resulting in dual therapy with LEV and LTG. During pregnancy, the seizure frequency remained at 1-2 per month. There were no generalized seizures. About six months ago, seizures began to occur more frequently, every other day or twice per week, and occasionally 4-5 times in one day.

Four months ago, the last modification of antiepileptic therapy was made: oxcarbazepine (OxCBZ) was introduced and titrated to 1200 mg/day, in addition to the existing LEV and LTG regimen. The patient underwent preoperative evaluation, but due to the lesion's proximity to language areas, surgical treatment was not indicated.

Four months after this last adjustment (the introduction of OxCBZ), a generalized skin rash developed, affecting the skin of the trunk, the abdomen, the back, and occasionally the forearms. The rash was macular in type, appeared intermittently with variable intensity, and was associated with marked erythema and itching. For this reason, the patient visited a dermatologist, who prescribed oral antihistamines, but the rash either did not resolve or only temporarily subsided and then reappeared. She was subsequently referred to an epileptologist to evaluate the possible role of antiepileptic drugs in causing the rash.

Diagnostic workup

Immediately upon admission, a neurological examination was performed, which was normal. Hematological laboratory tests were performed ​​​(Table 1).

**Table 1:** Hematological laboratory findings at admission Apart from the reduced hemoglobin and serum iron levels, all other laboratory findings were within normal limits

General hematology	Patient's value	Normal reference range
Red blood cells	4.3/L	3.9-5.2/L
Hemoglobin	106 g/L (low)	120-153 g/L
Hematocrit	0.35 L/L (low)	0.35-0.47 L/L
Erythrocyte sedimentation	6 mm/hour	1-10 mm/hour
White cell count	9.4 × 10^9^/L	4-10 × 10^9^/L
Neutrophil count	5.4 × 10^9^/L	2-7.5 × 10^9^/L
Lymphocyte	2.9 × 10^9^/L	0.8-4 × 10^9^/L
Monocyte	0.9 × 10^9^/L	0-1 × 10^9^/L
Eosinophil	0.05 × 10^9^/L	0-0.4 × 10^9^/L
Basophil	0.03 × 10^9^/L	0-0.1 × 10^9^/L
Platelets	371 × 10^9^/L	140-400 × 10^9^/L

 Biochemical laboratory tests were performed (Table 2).

**Table 2:** Biochemical laboratory findings at admission Except for elevated levels of total cholesterol, low-density lipoprotein (LDL) cholesterol, and gamma-glutamyl transferase (GGT), all other biochemical parameters were within normal reference ranges

General biochemistry	Patient's value	Normal reference range
Urea	4.4 mmol/L	2.7-8.08 mmol/L
Creatinine	75 µmol/L	44-80 µmol/L
Sodium (serum)	138 mmol/L	136-145 mmol/L
Potassium (serum)	4.6 mmol/L	3.5-5.1 mmol/L
Glucose (serum)	4.4 mmol/L	4.1-5.8 mmol/L
Total protein (serum)	78.5 g/L	64-83 g/L
Alkaline phosphatase (serum)	101 U/L	35-104 U/L
Alanine aminotransferase	22.2 U/L	<33 U/L
Aspartate aminotransferase	21.2 U/L	<32 U/L
Total bilirubin (serum)	4 µmol/L	0-21 µmol/L
Amylase (serum)	61 U/L	28-100 U/L
Gamma-glutamyl transferase	94 U/L (high)	<40 U/L
Calcium	2.32 mmol/L	2.15-2.5 mmol/L
Cholesterol (serum)	8.03 mmol/L (high)	<5.2 mmol/L
Low-density lipoprotein cholesterol (serum)	5.48 mmol/L (high)	<2.59 mmol/l
High-density lipoprotein cholesterol	1.93 mmol/L	>1.68 mmol/L
Triglycerides (serum)	1.37 mmol/L	<1.7 mmol/L
C-reactive protein (serum)	0-73 mg/L	<5 mg/L
Iron serum	5.8 µmol/L (low)	5.83-34.5 µmol/L

**Figure 1:**
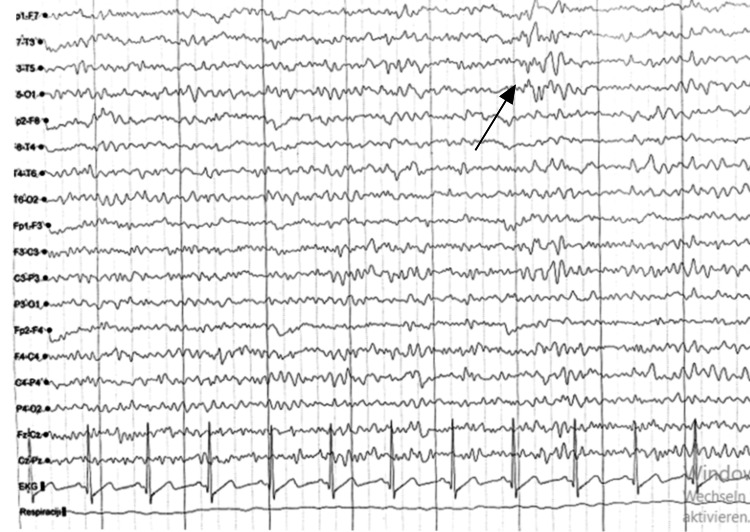
EEG showing left temporal rhythmic slowing and sharp wave discharges These findings are consistent with focal electrocortical dysfunction

A standard electroencephalographic recording was performed for 20 minutes (Figure 1).

**Figure 2:**
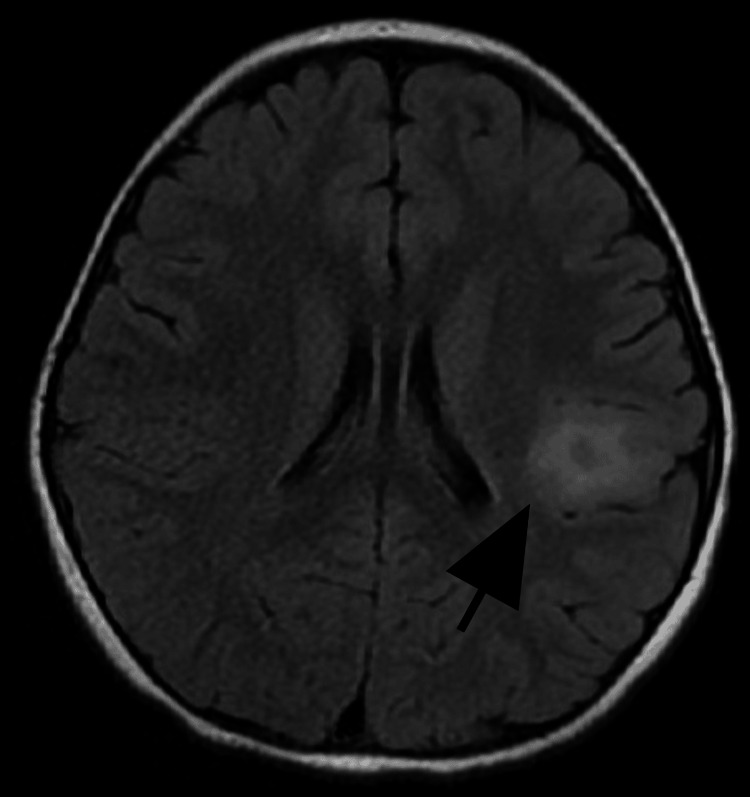
Brain MRI (axial FLAIR) Subtle T2/FLAIR hyperintensity in cortical grey matter. No diffusion restriction and normal sulcal/gyral patterns MRI, magnetic resonance imaging; FLAIR, fluid-attenuated inversion recovery

**Figure 3:**
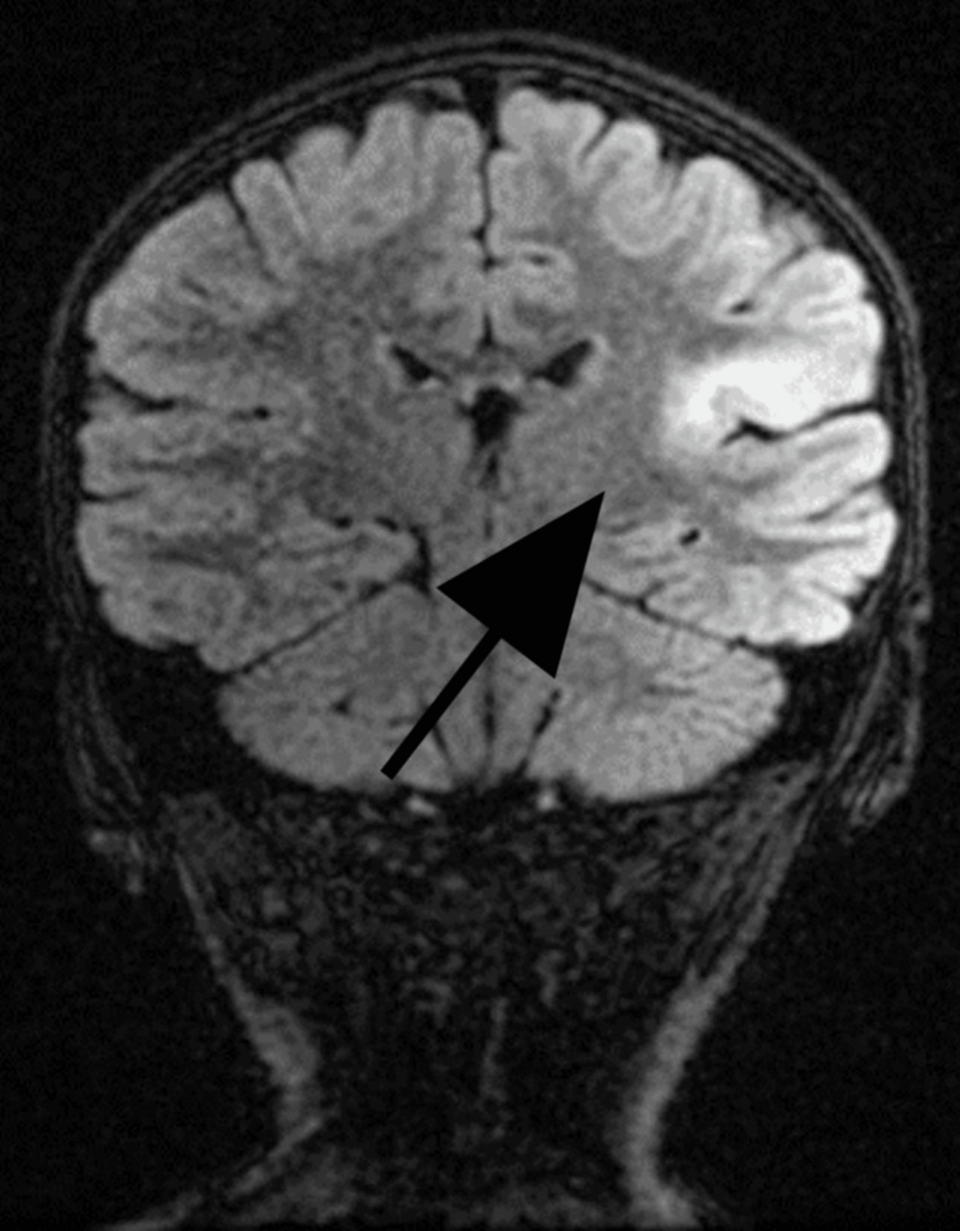
Brain MRI (coronal FLAIR) Cortical thickening and blurring of the grey-white junction in the left parietal cortex and T2/FLAIR hyperintensity of subcortical white matter with a transmantle sign MRI, magnetic resonance imaging; FLAIR, fluid-attenuated inversion recovery

A magnetic resonance imaging (MRI) of the brain was performed on a 1.5 T machine (Figures 2, 3).

**Figure 4:**
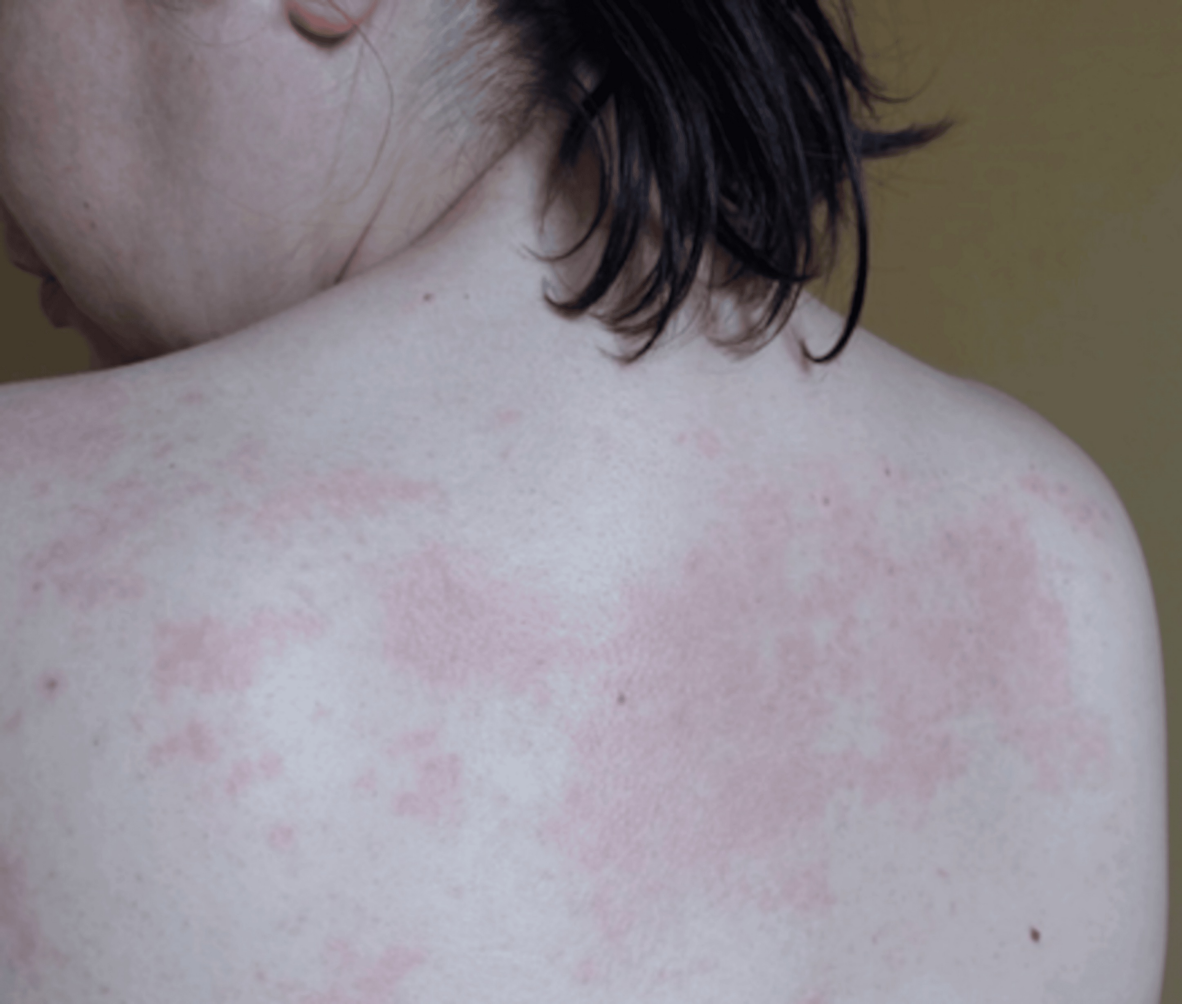
Generalized maculopapular rash affecting the upper back Maculopapular rash and erythematous changes involved a large portion of the skin

At hospital admission, the patient presented with generalized skin changes in the form of erythematous and macular lesions, most prominent on the skin of the back, abdomen, and extremities (Figure 4).

**Figure 5:**
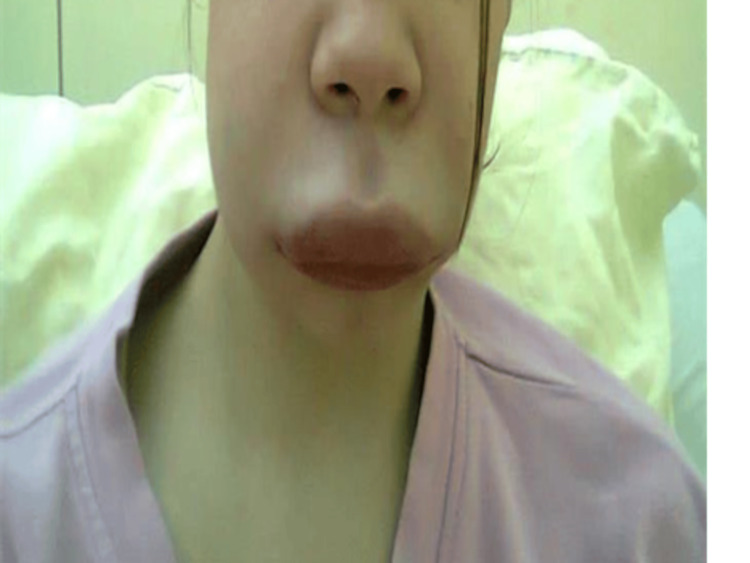
Lip angioedema in a patient treated with multiple antiepileptic drugs Angioedema occurred intermittently, mostly on days when corticosteroids were not administered

On the second day of hospitalization, the patient experienced an episode of angioedema, presenting as swelling of the lips and tongue, accompanied by mild dyspnea (Figure 5). This was the patient's first episode of angioedema in her medical history. The episode was successfully managed with parenteral corticosteroids and antihistamine therapy. Episodes of angioedema recurred intermittently during hospitalization on days when corticosteroids were not administered.

Management and outcomes

Since the patient had no prior allergic reactions to antiepileptic drugs or medications in general, OxCBZ was initially suspected as the cause of the allergic reaction, as it was the most recently added drug. The therapy was gradually discontinued over five days, with the intermittent administration of parenteral midazolam during seizure episodes. Oral antihistamine therapy with desloratadine was started at the same time.

On the second day of hospitalization, the patient developed lip and tongue edema accompanied by an episode of choking. Parenteral corticosteroid therapy with Lemod-Solu (1 mg/kg) was administered, which led to the resolution of the edema. Despite the discontinuation of oxcarbazepine, the skin rash persisted with slightly reduced intensity and a fluctuating appearance. On days when angioedema reappeared, corticosteroids were administered intermittently.

It was decided to gradually discontinue LTG, an antiepileptic drug frequently associated with allergic reactions, and to begin replacing it with lacosamide (LCZ). Over the following 10 days, LTG was completely discontinued and replaced with LCZ, which was gradually titrated. The rash persisted, with occasional episodes of angioedema. During this period, parenteral antihistamines were used daily, while corticosteroids were administered intermittently, every other day.

The seizure frequency did not worsen during this time: the patient experienced 2-3 seizures in 10 days, which was consistent with her previous frequency.

Finally, LEV was discontinued for one week, while zonisamide (ZON) was introduced in parallel. Following the complete discontinuation of LEV, the skin changes resolved, and by the fifth day after stopping LEV, the patient no longer exhibited any dermatological symptoms.

The patient's seizure frequency stabilized at approximately two focal seizures per week, which was consistent with her baseline level prior to recent medication changes. LEV was therefore identified as the most likely cause of the hypersensitivity reaction.

## Discussion

The incidence of cutaneous eruptions during antiepileptic drug (AED) therapy is estimated to range from 1.7% to 8.8%. Milder reactions, such as maculopapular and morbilliform rashes, most commonly occur between five days and eight weeks after the initiation of treatment. These rashes typically affect the face and body, are not accompanied by swelling, and usually resolve after the discontinuation of the medication [1].

On the other hand, more serious but considerably rarer skin reactions include drug reaction with eosinophilia and systemic symptoms (DRESS) syndrome, erythema multiforme (EM), Stevens-Johnson syndrome (SJS), and toxic epidermal necrolysis (TEN). One of the most severe adverse effects associated with AEDs is DRESS syndrome, which can appear several weeks after starting therapy and is characterized by systemic symptoms such as fever, edema, rash, and internal organ involvement [6]. Although this reaction is most commonly linked to aromatic AEDs such as phenytoin and carbamazepine, it has also been reported in patients receiving nonaromatic drugs, including LEV [2,4].

EM, SJS, and TEN can present with varying degrees of severity within the same clinical spectrum, with EM representing the mildest form. While EM may occur shortly after the administration of intravenous LEV [7], typically within 24-48 hours of drug exposure, SJS and TEN usually manifest between five and 28 days after initiation [4,5].

The hypersensitivity mechanism to AEDs is primarily attributed to the action of toxic metabolites that either directly damage cells or act as prohaptens, triggering a T-cell-mediated immune response. Morbilliform reactions are mainly associated with cluster of differentiation 4+ (CD4+) T cells, whereas SJS and TEN are characterized by a predominance of CD8+ T cells. In contrast, DRESS syndrome is accompanied by nonspecific, heightened T-cell activity [1].

There are four main theories that explain the mechanisms of T-cell activation by drugs: the hapten hypothesis, the direct interaction of drug metabolites with major histocompatibility complex (MHC) molecules, the pharmacological interaction (PI) concept without metabolic activation (the PI concept), and the alteration of the peptide repertoire on the surface of antigen-presenting cells.

IgE-mediated reactions are rare but have been reported with aromatic AEDs, while reactive metabolites, such as those produced by carbamazepine, phenytoin, valproate, and lamotrigine, can directly activate T cells [4,5].

Genetic predisposition associated with human leukocyte antigen (HLA) class I and II alleles is considered to play an important role in the pathogenesis of dermatological reactions [8]. Most allergic reactions caused by AEDs are the result of delayed, T-cell-mediated hypersensitivity. These reactions often have an insidious onset and may appear several weeks after initiating a new treatment, making it particularly challenging to identify the causative drug in patients receiving multiple medications. This suggests that T-cell reactivity may be directly induced by the drug itself, likely involving HLA class I proteins and, in some cases, class II [8,9].

Regarding delayed hypersensitivity, it has been observed that even chemically inert drugs can induce hypersensitivity reactions, which led to the development of the "PI concept" (pharmacological interaction with immune receptors). This concept describes a direct and reversible interaction of the drug with T-cell receptors (TCR) or MHC molecules. Depending on the type of drug and the patient's genetic predisposition (e.g., HLA-B*5701 in the case of abacavir), the drug may first bind either to the TCR or to the MHC, subsequently triggering the activation of specific T cells [10].

Although levetiracetam is generally considered safer than other AEDs with regard to cutaneous reactions, cases of maculopapular and morbilliform rashes have been reported in 1%-2% of patients. In general, patients presenting with uncomplicated maculopapular eruptions without systemic symptoms or abnormal laboratory findings tend not to progress to more severe syndromes, even if the offending drug is continued [3,9].

According to the literature, cutaneous adverse reactions typically occur within the first few days or up to several months after initiating LEV. Bhoi et al. reported a case of generalized maculopapular rash with pruritus affecting 70% of the body surface, which developed 10 days after starting levetiracetam [11]. More severe reactions such as SJS and TEN are extremely rare but have been documented, most often occurring within eight weeks of treatment initiation [3,12].

This time frame, ranging from a few days to two months, makes identifying the causative agent more challenging, especially in patients receiving multiple medications.

In our case, the patient developed a generalized skin rash eight months after starting LEV, which is highly unusual. A review of the available literature did not reveal any previously reported cases of such a delayed dermatological reaction to LEV.

Angioedema induced by LEV is extremely rare but has been reported in the literature, typically within the first few weeks of therapy. It can represent a medical emergency requiring the immediate discontinuation of the drug and the administration of antihistamines and corticosteroids [5,6].

Our patient also experienced an episode of angioedema, which further complicated the clinical picture and prolonged hospitalization. In this case, both angioedema and the skin rash occurred in a delayed fashion.

There was no evidence of cross-reactivity in our patient, considering that during the early phase of illness, they had already received CBZ and later LTG without any adverse reactions. It is well known that cross-reactivity between CBZ and OxCBZ can be as high as 70% and around 20% among aromatic AEDs (such as phenytoin, phenobarbital, CBZ, OxCBZ, and LTG) [5].

Fortunately, there were no signs of DRESS syndrome in our patient. DRESS syndrome includes systemic manifestations such as lymphadenopathy, elevated liver enzymes, interstitial nephritis, and hematological abnormalities (eosinophilia and atypical lymphocytosis). It is most commonly triggered by phenytoin and carbamazepine. Although the diagnosis is primarily clinical, lymphocyte transformation testing and intradermal testing may be helpful in unclear cases [13].

Angioedema, as an IgE-mediated type 1 hypersensitivity reaction, clinically resembles urticaria but involves deeper swelling of the dermis and submucosa. It can lead to life-threatening airway obstruction due to laryngeal edema. The mechanism involves allergen binding to IgE molecules on basophils and mast cells, leading to the release of inflammatory mediators [5,9].

The analysis of the FDA Adverse Event Reporting System revealed that AEDs are associated with a significantly higher risk of SJS and TEN, up to nine times greater than non-AEDs, with certain drugs such as ZON, LTG, and phenytoin showing especially elevated risk, underscoring the importance of clinician and patient awareness for early recognition and prevention [14]. In the present case, rash and angioedema occurred during triple therapy with LEV, LTG, and OxCBZ. Initially, OxCBZ was suspected as the cause, since it had recently been introduced. LTG is also known to cause skin reactions in approximately 8% of patients, with significantly increased risk when combined with valproate, though this was not a factor in our case [5].

However, symptoms persisted even after the discontinuation of OxCBZ and LTG. Only after the withdrawal of LEV did the symptoms completely resolve, indicating its likely role as the causative agent. Although LEV is generally considered safe in terms of dermatological adverse effects, increasing reports of urticaria, angioedema, and even SJS have been documented. Due to the rarity of such reactions, it is possible that they are underrecognized in clinical practice [4,9].

LEV can cause both skin reactions and angioedema in a delayed fashion, even several months after treatment initiation, and these reactions may fully resolve after drug withdrawal and appropriate treatment (including the parenteral administration of antihistamines and corticosteroids) [14].

Although this report describes a single patient, the case highlights important diagnostic and therapeutic challenges associated with managing cutaneous adverse reactions in polytherapy settings. The delayed onset of symptoms, particularly with levetiracetam, a drug generally considered to have a favorable safety profile, underscores the need for continued clinical vigilance, even months after initiating therapy. This case illustrates the importance of considering all concurrent medications when evaluating adverse reactions and reinforces the value of sequential drug withdrawal in identifying the causative agent. Moreover, the absence of access to pharmacogenetic testing in certain healthcare systems, such as ours, limits the ability to predict allergic susceptibility, further complicating personalized AED selection. These limitations emphasize the need for clinical awareness, detailed monitoring, and a patient-centered approach in treatment planning.

In patients with poorly controlled epileptic seizures who are on polytherapy and simultaneously experience adverse drug reactions, prolonged hospitalization and the need to adjust existing AED regimens can further increase anxiety levels.

The gold standard for identifying specific drug hypersensitivity is drug provocation testing. However, for certain severe reactions such as SJS and TEN, provocation testing is extremely risky due to the potential for the reactivation of mucocutaneous inflammation and blister formation.

In cases where there is suspicion about the cause of type 1 hypersensitivity reactions or morbilliform rashes and skin prick tests (SPT) were negative or inconclusive, graded drug challenges may be performed at specific time intervals. For type 1 reactions, the recommended dosing schedule starts with 0.1% of the therapeutic dose, followed by 1% three hours later, then 10% after another three hours, and finally 100% three hours after that. It is essential that intravenous access and emergency facilities are readily available during testing.

For delayed-onset morbilliform rashes, the same dosing schedule may be given with 24-hour intervals between doses, allowing the patient to return home between exposures.

As an alternative to drug provocation in patients with mild type 1 reactions without cardiorespiratory compromise or mucocutaneous involvement, active desensitization protocols may be considered. Desensitization has proven successful in managing delayed drug hypersensitivity reactions, particularly in cases of mild exanthems, where clinical tolerance is achieved through the gradual reintroduction of the offending drug, with success rates varying depending on the clinical presentation, drug involved, and protocol used [15].

## Conclusions

Levetiracetam is generally well tolerated but can cause delayed-onset cutaneous adverse reactions, including angioedema, which may be life-threatening. Clinicians should closely monitor patients using levetiracetam, whether as monotherapy or as part of polytherapy, and discontinue the drug at the first signs of a rash unless another cause is clearly identified. Early recognition and prompt intervention are key to preventing serious complications.
